# Vaccination of older adults: Influenza, pneumococcal disease, herpes zoster, COVID-19 and beyond

**DOI:** 10.1186/s12979-021-00249-6

**Published:** 2021-10-09

**Authors:** Birgit Weinberger

**Affiliations:** Institute for Biomedical Aging Research, Universität Innsbruck, Rennweg 10, 6020 Innsbruck, Austria

**Keywords:** Vaccination, Vaccine, Age, Elderly, Influenza, Streptococcus pneumoniae, Herpes zoster, COVID-19, SARS-CoV-2

## Abstract

Preserving good health in old age is of utmost importance to alleviate societal, economic and health care-related challenges caused by an aging society. The prevalence and severity of many infectious diseases is higher in older adults, and in addition to the acute disease, long-term sequelae, such as exacerbation of underlying chronic disease, onset of frailty or increased long-term care dependency, are frequent. Prevention of infections e.g. by vaccination is therefore an important measure to ensure healthy aging and preserve quality of life. Several vaccines are specifically recommended for older adults in many countries, and in the current SARS-CoV-2 pandemic older adults were among the first target groups for vaccination due to their high risk for severe disease. This review highlights clinical data on the influenza, *Streptococcus pneumoniae* and herpes zoster vaccines, summarizes recent developments to improve vaccine efficacy, such as the use of adjuvants or higher antigen dose for influenza, and gives an overview of SARS-CoV-2 vaccine development for older adults. Substantial research is ongoing to further improve vaccines, e.g. by developing universal influenza and pneumococcal vaccines to overcome the limitations of the current strain-specific vaccines, and to develop novel vaccines against pathogens, which cause considerable morbidity and mortality in older adults, but for which no vaccines are currently available. In addition, we need to improve uptake of the existing vaccines and increase awareness for life-long vaccination in order to provide optimal protection for the vulnerable older age group.

Low birth rates and higher life expectancy are transforming the age pyramid in Europe towards a much older population. Between 2010 and 2020 the share of the population older than 65 years of age increased by 3 percentage points in the EU (from 17.6 to 20.6%) and is projected to further increase to more than 31% by 2100. The share of those older than 80 years is projected to have a 2.5-fold increase (5.9% to 14.6%) from now until 2100 [1]. This leads to challenges of societies, economies and health care systems on many levels. Preserving good health in old age is of utmost importance to alleviate some of these challenges, but of course also for the well-being and quality of life for every individual person. This review focusses on vaccines to prevent infectious disease in older adults. The incidence of many infections is higher in older compared to younger adults [2] and morbidity as well as mortality due to infectious diseases is increased in this age group. In addition to the immediate impact of the disease many older persons do not recover fully after an acute episode of infection and chronic co-morbidities might be exacerbated. These phenomena have been described for influenza, pneumococcal disease and herpes zoster and can lead to long-term sequelae such as onset or increase of frailty, impairments in activities of daily living and even loss of independence [3–5]. Prevention of infections e.g. by vaccination is therefore an important measure to ensure healthy aging and preserve quality of life.

## Influenza

Influenza causes approximately 15,000-70,000 deaths annually in Europe, mainly in older adults [6], and complications such as exacerbations of underlying pulmonary disease or bacterial co-infections further increase the burden of disease [5, 7]. Vaccination against influenza is recommended in many countries for everybody, but is particularly relevant for older adults and other risk groups. Historically, influenza vaccines (TIV, trivalent influenza vaccine) contained two influenza A strains (H1N1 and H3N2) and one B strain (Yamagata or Victoria lineage). Influenza viruses are highly variable with antigenic shift (exchange of RNA segments leading to novel strains, e.g. H5N1) leading to pandemics and antigenic drift (point mutations) necessitating annual adjustments of the seasonal vaccines. Based on world-wide surveillance data WHO defines the exact composition for the annual vaccine each year. As both B lineages have co-circulated for several years, quadrivalent influenza vaccines (QIV) containing two A and two B strains have been developed and are now in widespread use [8]. Most influenza vaccines are produced by an egg-based manufacturing process, in which candidate vaccine viruses are propagated in fertilized hen´s eggs. Alternatively, influenza vaccine viruses can be grown in mammalian cell culture. Standard influenza vaccines are either split virus vaccines, which comprise disrupted viral envelopes, or subunit vaccines, for which the viral nucleocapsid is removed in further purification steps [9]. In addition, recombinant influenza vaccines use hemagglutinin proteins expressed in insect cells using a baculovirus system.

### Immunogenicity

Immunogenicity of standard TIV is lower in older compared to young adults [10] and frailty and co-morbidities further decrease vaccine-induced immune responses [11, 12]. It has also been reported that influenza-specific antibody titers decline faster in older persons leading to loss of seroprotection until the following season, or even towards the end of the same season for some influenza strains [13, 14]. Antibodies against the viral hemagglutinin are widely used as surrogate of protection in the context of influenza vaccination [15], but might not be an ideal measure in older adults, as vaccinees with low titers may be still protected and vice versa [16]. It has been shown that memory B cells and plasmablasts are retained in older adults, despite lower antibody titers compared to young adults even after repeated vaccination. Impaired differentiation from memory B cells towards plasma cells might be responsible for this phenomenon [17]. In addition to antibodies, cell-mediated immune responses are also important to combat influenza virus infection and T cell parameters (e.g. IFN-γ and IL-10 production, Granzyme B activity) might be better predictors of clinical protection [18–20].

### Clinical efficacy and effectiveness

In placebo-controlled trials enrolling young adults TIV is up to 70% effective against laboratory-confirmed influenza [21, 22], but lower efficacy has been observed in older adults [23, 24]. Meta-analysis of influenza vaccine immunogenicity, efficacy and effectiveness is difficult as different vaccine formulations are used (split vs. subunit), vaccine composition changes from year to year, clinical endpoints are variable (influenza-like illness, laboratory-confirmed influenza, hospitalization, etc.) and history of exposure (infection and prior vaccination) may vary between cohorts. Despite these uncertainties there is consensus that standard influenza vaccines are less immunogenic and efficient in older compared to younger adults and that improved vaccines for the older population are necessary.

### Strategies for improvement

An obvious strategy to improve immunogenicity of vaccines might be to increase the amount of antigen per dose. A higher antigen dose should result in increased uptake and presentation by antigen-presenting cells and therefore in stronger activation of adaptive immune cells. In the US, Canada, Brazil, Australia and the UK, a trivalent high-dose influenza vaccine has been used for several years (HD-TIV). This vaccine contained 60μg hemagglutinin for each strain instead of the standard 15μg. HD-TIV was shown to induce higher anti-hemagglutinin antibody concentrations and seroprotection rates, as well as increased numbers of influenza-specific T cells in older adults compared to TIV [25, 26]. Clinical efficacy of HD-TIV compared to standard TIV in older adults was summarized in meta-analyses, which reported a lower risk to develop laboratory-confirmed influenza (relative risk 0.76) [27] and a higher relative efficacy against pneumonia (rVE 24.3%), hospitalization for influenza (rVE 17.8%), and influenza-like illness (rVE 19.5%) [28].

An alternative strategy to improve vaccine-induced immune responses is the use of adjuvants in order to protect cohorts, which develop unsatisfactory immune responses after standard immunization (e.g. older adults). Aluminum salts have been used in human vaccines for approximately 100 years in combination with various, but not with influenza antigens. As one of the first modern adjuvants the oil-in-water emulsion MF59, which comprises squalene and the surfactants Tween 80 and Span85 was licensed as an adjuvant in seasonal influenza vaccines for older adults in 1997 (adjuvanted TIV, aTIV). It has also been approved for other age groups including children (6-24 months) and in pandemic influenza vaccines. MF59 induces proinflammatory chemokines and cytokines at the site of injection thereby recruiting innate immune cells and facilitating efficient antigen uptake, enhances differentiation of dendritic cells in the lymph nodes, and is involved in shaping the germinal center reaction [29, 30]. Antibody responses following aTIV are slightly higher compared to TIV (summarized in [31, 32]), and the CD4^+^ T cell response is elevated [33]. Interestingly, antibodies elicited by aTIV recognize drifted influenza strains more efficiently as antibodies against additional epitopes within the influenza hemagglutinin are induced [34, 35]. A meta-analysis reported greater efficacy of aTIV in preventing laboratory-confirmed influenza (adjusted odds ratio 0.37; 95%CI: 0.14–0.96) and hospitalizations due to pneumonia/influenza (adjusted risk ratio 0.75; 95% CI: 0.57–0.98) compared to standard TIV [36].

Clinical effectiveness of HD-TIV and aTIV were recently compared directly in two studies. A retrospective cohort study, which analyzed data of persons above 65 years during the 2016/2017 and the 2017/2018 influenza seasons reported that HD-TIV provided better protection from respiratory-related hospitalizations compared to aTIV, with a pooled relative vaccine effectiveness (rVE) of 12% (95% CI, 3.3%-20%) [37]. In contrast, in a similar study effectiveness against any influenza-related medical encounter was higher for the adjuvanted trivalent vaccine (rVE 7.7%; 95%CI: 2.3%-12.85) compared to the high-dose trivalent vaccine during the 2017/2018 influenza season. Similar results were obtained for the following season [38]. The challenges in comparing different studies investigating efficacy and/or effectiveness of influenza vaccines have been mentioned above.

Both high-dose and adjuvanted influenza vaccines have recently been modified to contain four instead of three different influenza strains (HD-QIV and aQIV) and have been licensed in many countries. Recent studies confirmed higher immunogenicity of HD-QIV compared to standard QIV [39] and non-inferiority to HD-TIV [40] as well as a satisfactory safety profile. Immunogenicity and safety of aQIV have been shown to be similar to aTIV in older adults [41].

Annual vaccination against influenza is recommended for all adults in many countries, but most recommendations emphasize the particular importance for vulnerable groups, such as older adults. Several countries specifically recommend the high-dose and/or adjuvanted influenza vaccines for older adults. In previous years these guidelines were complicated by the fact that these “improved” vaccines were only trivalent, whereas the standard vaccine had already been available in a quadrivalent formulation. For the season 2021/2022 HD-QIV and aQIV will be available in many countries and are included in specific national recommendations for older adults. As examples, Germany issued its first recommendation for a specific vaccine, namely the HD-QIV, for the 2021/2022 season [42]. In the UK, aTIV or HD-TIV was recommended for older adults during the 2020/2021 season [43], whereas for the 2021/2022 season aQIV or HD-QIV should be used [44]. In contrast, the Advisory Committee on Immunization Practices in the US did not recommend a specific vaccine formulation for the older population for 2020/2021 despite the fact that adjuvanted and high-dose quadrivalent formulations were expected to be available [45], and specific recommendations for 2021/2022 were not yet available at the time of publication.

### Future developments

Next-generation influenza vaccines are being developed. One approach is the use of alternative adjuvants, which might overcome the diminished responsiveness of the aged immune system. As mentioned above, MF59 is used successfully in influenza vaccines and other squalene-based adjuvants have also been tested (summarized in [46]). AS03 has been used in the pandemic influenza vaccine of 2009 and induced higher antibody concentrations and seroprotection levels in older adults compared to a whole-virus vaccine or a non-adjuvanted split vaccine [47, 48]. In both studies the amount of antigen in the adjuvanted vaccine was substantially lower than in the comparator vaccines. Superior clinical efficacy of seasonal TIV adjuvanted with AS03 compared to standard TIV could be demonstrated for protection against influenza A, and particularly A/H3N2 infection, but not against infection with any influenza strain. In addition this study showed higher efficacy against hospital admission for pneumonia and all-cause death in descriptive estimates [49]. AF03 (squalene-based emulsion), Advax-CpG55.2 (inulin + TLR9-agonist), GLA-SE (emulsion of TLR4-agonist) and several other TLR-agonists and alternative emulsions have been tested in combination with influenza antigens and there are many more potential adjuvants, such as e.g. cytokines, T cell stimulating adjuvants, DNA-based adjuvants etc., which might be good candidates to boost vaccine-induced immune responses in older adults, but have not yet been tested in this age group. More extensive reviews on adjuvants for influenza vaccines can be found elsewhere [30, 50]. After the rapid success of mRNA vaccines against COVID-19 (see below), several manufacturers started or intensified development of mRNA vaccines against influenza. The use of mRNA vaccines against influenza was first suggested in 2012 and it has been shown that this vaccine candidate offers protection from influenza in animal models including aged mice [51]. Compared to “traditional” influenza vaccine manufacturing in eggs or cell culture, mRNA vaccines could be produced much faster in large quantities. This is particularly relevant for influenza vaccines, as a later decision for a specific seasonal vaccine composition could improve the match between vaccine and circulating virus strains. In July 2021 Moderna started the first Phase I/II clinical trial with a seasonal influenza mRNA vaccine in humans (NCT04956575).

Influenza-specific antibodies show some degree of cross-reactivity towards related viral strains and influenza-specific T cells can recognize conserved epitopes. However, clinical data show that a mismatch between vaccine strains and circulating virus strains decreases efficacy and effectiveness of influenza vaccines. Overall influenza vaccine effectiveness is usually 40-60% in years with adequate matching of vaccine and circulating virus strains [52]. But as an example, in the 2014/2015 season vaccine effectiveness was only 19% against influenza and only 6% against H3N2 strains [52]. The circulating influenza A H3N2 strains had changed significantly (antigenic drift) after selection of the vaccine strains [53] leading to a pronounced mismatch in this year. Similar effects were observed in the seasons 2004/2005 and 2005/2006 [54]. Adjuvants can improve the production of cross-reactive antibodies which recognize drifted strains (summarized in [55]), but universal influenza vaccines would be desirable to address the immense antigenic variability of influenza viruses and to be prepared for novel, potentially pandemic influenza strains. Various approaches, such as antigens based on the conserved stem-region of hemagglutinin, chimeric hemagglutinin proteins, peptides and nucleic acid platforms, are currently tested in clinical trials. While cross-reactive or broadly reactive antibodies contribute to a broader or potentially universal protection, T cells also play a crucial role. CD4^+^ T cells contribute to an efficient immune response in several ways. They have direct effector functions e.g. cytokine secretion and cytolysis in the lung, are important for rapid innate responses and provide help to B cells, which ensures optimal antibody production, as well as to CD8^+^ T cells. The cytotoxic CD8^+^ T cells recognize and eliminate infected cells, thereby limiting virus spread in the body. Many of the vaccine candidates for universal influenza vaccines utilize antigens and/or technologies, which aim to elicit robust T cell responses. A comprehensive summary of these approaches has recently been published [56]. It is very likely that adjuvants and/or vaccine-delivery platforms will be essential for optimal vaccine responses, particularly in the older population [57–59].

## Pneumococcal disease

*Streptococcus pneumoniae* (pneumococcus) can be classified into more than 90 distinct serotypes based on their polysaccharide capsule of which only a limited number are pathogenic [60]. Antimicrobial resistance of *S. pneumoniae* is an increasing problem [61]. Clinical presentation of *S. pneumoniae* infection can be non-invasive (otitis media, sinusitis, conjunctivitis, pneumonia) or invasive (bacteremic pneumonia, meningitis, sepsis). Incidence of invasive pneumococcal disease (IPD) as well as pneumococcal pneumonia increases with age. *S. pneumoniae* is the most frequently isolated pathogen causing community-acquired pneumonia (CAP) in older adults. In the US nearly 30,000 cases of invasive pneumococcal disease (IPD) and over 500,000 cases of non-bacteremic pneumococcal pneumonia were estimated to occur every year in persons older than 50 years, resulting in more than 25,000 pneumococcus-related deaths [62]. Bacterial co- or secondary infections are frequently observed in influenza patients. The exact numbers of co-infections vary greatly in different studies; a meta-analysis reported bacterial infections in 11% to 35% of influenza patients with *S. pneumoniae* being the most common pathogen accounting for 35% (95% CI: 14%-56%) of all bacterial co-infections [63].

Two types of vaccines are available against *S. pneumoniae*; polysaccharide vaccines (PPV), which contain the purified bacterial capsule polysaccharides, and conjugated vaccines (PCV), for which the polysaccharides are conjugated to carrier proteins. The serotype coverage of the different vaccines is summarized in Table 1.

**Table 1:**
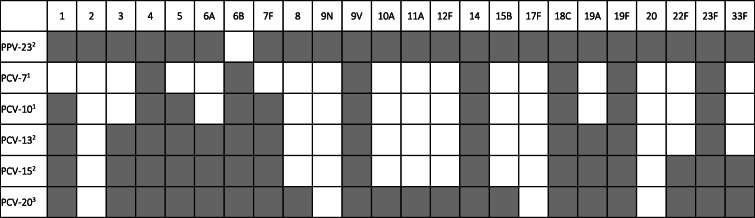
Serotypes included in pneumococcal polysaccharide (PPV) and conjugate (PCV) vaccines

^1^ licensed, but not for older adults

^2^ licensed for older adults

^3^ in development

Purified polysaccharides are T cell-independent antigens, and as such elicit a distinct immune response. Without T cell help, B cell activation and differentiation happen independently of germinal centers resulting in short-term antibody production, a lack of B cell memory and mainly IgM and IgG_2_ responses [64, 65]. Infants are not able to mount efficient immune responses against polysaccharide antigens in the first two years of life. Therefore, the PPV-23 vaccine is not suitable for young children and has only been licensed for adults despite the fact that non-invasive and invasive pneumococcal disease is highly prevalent in infants. PPV-23 has been recommended and utilized in many countries for the older population for several decades. Carrier proteins, which are chemically conjugated to the polysaccharides facilitate T cell-dependent immune responses where carrier-specific T cells provide T cell help for polysaccharide-specific B cells [65]. Class switch and avidity maturation can take place, memory B cells are generated, and the conjugate vaccines (PCV) are immunogenic in infants. PCV-7 was introduced in the late 1990s/early 2000s for young children, but was not licensed for adults. The incidence of IPD in the target age group declined substantially after introduction of this vaccine. As PCV does not only prevent disease, but also carriage of *S. pneumoniae*, the incidence of IPD also decreased in older adults, presumably due to reduced transmission from children to older adults [66]. A certain degree of serotype replacement was observed in all age groups in the following years leading to the development of PCV-10 and PCV-13, which contain additional serotypes and replaced PCV-7 in the childhood vaccination programs of many countries. PCV-13 was the first PCV, which was also licensed for adults and is now used in many countries for the older population. Serotype replacement can still be observed and development of PCVs containing even more serotypes is ongoing (see below).

### Immunogenicity

Two different methods are used to quantify polysaccharide-specific antibodies and to determine immunogenicity of the pneumococcal vaccines. ELISA-based methods detect IgG antibodies but no other antibody classes and early ELISA methods were often unspecific. Improved protocols solved this problem, but some older studies need to be interpreted with caution [67]. Functional antibodies can be measured by opsonophagocytosis assays (OPA). In young children, these two assays show a strong correlation, but this is not the case in older adults and immunocompromised patients [68]. It has been concluded from several studies [69–73] that OPA-measurements are probably a more reliable correlate of protection and that they should be utilized despite the fact that they are more complex and expensive and less standardized than ELISA methods.

With age, immunogenicity of PPV-23 decreases, as shown by reduced opsonophagocytic activity, alterations in class and subclass usage as well as in somatic hypermutation [74–76]. Currently, PPV-23 and PCV-13 are available for older adults, but many studies comparing immune responses to PPV and PCV in this age group were already performed with PCV-7. Some studies reported higher antibody levels (ELISA and OPA) for PCV-7 [77], whereas others did not detect significant differences between antibodies elicited by PCV-7 or PPV-23, respectively [78–80]. This might be explained by the fact that the patient populations were heterogenous, most studies were relatively small and previous vaccination with PPV-23 impacts the response to PCV-7. Frailty further impairs antibody responses after pneumococcal vaccination [78]. More recent studies compare PPV-23 and PCV-13 and systematic meta-analyses show higher antibody levels for the majority of serotypes and non-inferiority for the others after vaccination with PCV-13 [81, 82]. Early studies showed that a second dose of PCV-7 one year after either PPV-23 or PCV-7 might be beneficial for antibody responses and particularly for their long-term maintenance [77, 79] and a more recent meta-analysis concluded that prior vaccination with PPV-23 did not influence the immunological response to PCV-13 [81].

### Clinical efficacy and effectiveness

Efficacy and effectiveness of pneumococcal vaccines have been mainly studied for IPD and in some studies for pneumococcal pneumonia. In an extensive meta-analysis pooled vaccine efficacy/effectiveness (VE) of PPV-23 against IPD and pneumococcal pneumonia was calculated for different types of studies and ranged from 73% against IPD in clinical trials to below 50 % in cohort studies (Table 2). The authors suspected a high risk of bias in the diagnosis of pneumococcal pneumonia for several studies, which they excluded from their analysis. However, these studies had been included in previous meta-analyses which failed to demonstrate a protective effect against pneumonia [84–87]. Clinical efficacy of PCV-13 was tested in a large Phase IV randomized, placebo-controlled trial including more than 84,000 older adults. In the per-protocol analysis PCV-13 was 45.6% (95% CI: 21.8-62.5%) effective against first episodes of community-acquired pneumonia (CAP) caused by vaccine-type strains and 75.0% (95% CI: 41.1-90.8%), against vaccine-type IPD, respectively [88]. It is still controversially discussed which pneumococcal vaccination strategy provides optimal protection against disease, as data on clinical efficacy of schedules combining PPV and PCV or repeated doses of either vaccine are lacking. Antibody responses to PCV-13 are stronger and can potentially be boostered by additional doses, but due to high vaccination rates in the pediatric population and the accompanying herd immunity effects, prevalence of the PCV-13 serotypes in older adults decreases and other serotypes might become more clinically relevant. PPV-23 covers additional serotypes, but there are concerns that PPV might induce tolerance or hyporesponsiveness upon repeated vaccination, similar to the meningococcal polysaccharide vaccine [89]. Vaccination recommendations are very heterogenous in Europe, where various countries recommend either PCV-13 or PPV-23, or PCV-13 followed by PPV-23. This combination aims to exploit the advantages of both vaccines. The sequential use of both vaccines was also recommended in the US for several years, but since 2019 only PPV-23 is generally recommended and the addition of PCV-13 should be considered for the individual patient in a shared decision process [90]. The uncertainties and inconsistencies in national recommendations might contribute to the fact that vaccination coverage for pneumococcal vaccine is still low in many countries [91–94].

**Table 2:** Pooled vaccine efficacy/effectiveness (VE) of PPV-23 against IPD and pneumococcal pneumonia (data from [83]).

	clinical trials VE [%] (95%CI)	cohort studies	case-control studies
IPD	73% (10-92)	45% (15-65)	59% (35-74)
pneumococcal pneumonia	64% (32-80)	48% (25-63)	n.d.

n.d. not done

### Strategies for improvement and future developments

As mentioned above development of next-generation conjugated vaccines containing additional serotypes is ongoing. Early stage clinical trials showed that safety and immunogenicity of a 15-valent and a 20-valent conjugated vaccine (Table 1) were similar to that of PCV-13 in older adults [95, 96]. PCV-15 has been tested in Phase III trials in adults over 50 years of age and younger adults with various risk factors (NCT03950622, NCT03480802, NCT03950856, NCT03615482, NCT03547167, NCT03615482) demonstrating immunogenicity when administered alone or concomitantly with influenza vaccine or followed by the 23-valent polysaccharide vaccine and has very recently been licensed in the US [97]. Phase III trials for PCV-20 are ongoing in older adults (NCT03835975, NCT04875533, NCT03760146, NCT04526574). As serotype prevalence differs between children and older adults, partially due to serotype replacement processes, it might be advisable to consider the option of including different serotypes in vaccines for the different age groups.

The use of adjuvants has been successful for various protein-based vaccines, but has not yet moved beyond early development for polysaccharide or conjugate vaccines. Alum does not improve immune responses to T-cell independent antigens or conjugates [98, 99], and various TLR agonists failed to increase antibody responses when co-administered with pneumococcal polysaccharides [100]. Several adjuvants, such as IC31 (TLR-9 agonist + antibacterial peptide) or a combination of MPL and synthetic cord factor formulated as an oil-in water emulsion showed promising results in mouse models [101, 102], but have not yet been tested clinically.

Universal vaccines against *S. pneumoniae* would be needed to fully overcome the risk of serotype replacement. A whole-cell vaccine candidate and various individual protein or peptide vaccines, most of them utilizing pneumococcal histidine triad protein D (PhtD), detoxified pneumolysin derivative (PlyD) and pneumococcal surface protein (PspA) or combinations of those are developed. Many of these vaccine candidates combine the antigens with different adjuvants. Several of these vaccine candidates show promising immunogenicity and safety profiles in early clinical studies and even more are still in pre-clinical development [103–111].

## Herpes zoster

Primary infection with varicella-zoster virus (VZV) usually occurs in childhood and manifests as chickenpox (varicella). Life-long viral latency is established in sensory ganglia, and reactivation of VZV, which can occur throughout life, is usually controlled by T cell responses (cell-mediated immunity, CMI) and therefore asymptomatic. In situations with reduced CMI, e.g. under immunosuppression or with increasing age, reactivations can manifest as herpes zoster (HZ) if the virus spreads through the sensory nerve to the corresponding dermatome. This results in a typically unilateral, frequently painful, segmented skin rash. A substantial increase of the HZ incidence with age (2/1,000 person-years at age 50; 6–8/1,000 person-years at 60; 8–12/1,000 person-years at age 80) was reported in a systematic review, which included 130 studies from various countries [112].

Pain occurring or persisting more than 3 months after onset of the rash is referred to as post-herpetic neuralgia (PHN), and is a frequent complication of HZ. PHN is often associated with severe pain, which is very difficult to manage therapeutically and can last for several months resulting in considerable impact on activities of daily living and quality of life [113, 114]. The incidence of PHN also increases with age from 18% in HZ patients older than 50 years to 33% in HZ patients older than 80 years [115]. HZ and PHN are prominent examples how an acute episode of infection can lead to long-term sequelae including loss of independence and institutionalization. Vaccination against HZ aims to restore the VZV-specific immune response, which was generated during the primary infection, in order to prevent the clinical consequences of viral reactivation.

A live-attenuated vaccine based on the Oka Merck virus strain is available to prevent primary infection with VZV in children, and the same strain (14-fold higher dose) was also used in older adults to prevent HZ. As a live-attenuated vaccine, it is not suitable for immunocompromised patients, who are at high risk for HZ, but it has a favorable reactogenicity and safety profile in immunocompetent persons including older adults. A second-generation vaccine against HZ contains the viral envelope glycoprotein E (gE) and the adjuvant system AS01B, which consists of 3-O-desacyl-4´-monophosphoryl lipid A (MPL, a derivative of lipopolysaccharide) and QS-21, a saponin found in the bark of the tree Quillaja Saponaria, formulated in liposomes, which act as antigen delivery systems. This combination enables uptake of the antigen via endocytosis, activates innate immune cells via the TLR-4 pathway and targets subcapsular macrophages in the lymph node [116–120], leading to efficient activation of adaptive immune responses [121]. The adjuvanted vaccine was licensed and is now used in Europe, Canada, the US, Japan and many other countries.

### Immunogenicity

Both vaccines induce antibody and cellular immune responses, but T cell mediated immunity (CMI) is considered to be essential for protection against herpes zoster, whereas only a minor role is attributed to antibodies. Immunogenicity was investigated in sub-cohorts of the pivotal Phase III trials for both vaccines. It has been shown that baseline VZV-specific T cell responses decline with age and are lower for persons ≥70 compared to those 60-69 years of age, whereas baseline antibody levels are independent of age and did not correlate with CMI [122]. Upon vaccination with the live-attenuated vaccine VZV-specific T cell responses increase and although the magnitude of these responses decreases over time, particularly within the first year, CMI remains above baseline levels for the observation period of three years. Similar observations were made for VZV-specific antibodies. VZV-CMI is significantly lower in subjects ≥70 compared to the 60-69 years-old cohort, but no age-related differences in antibody responses were observed. Despite an inverse correlation of VZV-CMI with the likelihood of developing HZ this study was not able to identify a surrogate marker or threshold level of protection [122]. Upon vaccination with two doses of the recombinant adjuvanted vaccine VZV-CMI (measured by gE-specific CD4^+^ T cells producing at least two cytokines) increases in more than 90% of the recipients and remains above the CMI-response threshold in 57% three years later. Specific CD4^+^ T cells persist substantially above pre-vaccination values even 9 years after vaccination [123]. A slightly lower proportion of vaccine recipients ≥70 remains above this threshold compared to younger participants confirming previous data on a moderate age-associated decline of T cell, but not antibody responses [124–126]. CD8^+^ T cell responses were rarely detected in this study. Antibody levels also increase substantially and remain over baseline for at least three years. Only minimal differences were observed between age groups at any time point. In contrast to the study with the live-attenuated vaccine, a moderate positive correlation between humoral and CMI responses was observed after vaccination with the recombinant vaccine [127]. Direct comparisons of T cell and antibody responses induced by the two vaccines are complicated by the fact that the live-attenuated vaccine contains a multitude of viral antigens whereas the recombinant vaccine includes only the glycoprotein gE. gE-specific CD4^+^ T cell responses are substantially higher following vaccination with the recombinant vaccine, but even total VZV-specific T cell responses were not superior following vaccination with the live-attenuated vaccine. In addition, the recombinant vaccine induced preferentially memory, but less effector T cells. The authors of this study conclude that gE-specific memory Th1-responses are relevant for protection and that high peak memory T cell responses are necessary for long-term persistence [128]. Antibody responses measured by ELISA are also higher after vaccination with the recombinant vaccine. Interestingly, the avidity of gE-specific antibodies at baseline (i.e. before vaccination) is much lower compared to antibodies specific for all VZV-glycoproteins and vaccination with the recombinant, but not the live-attenuated vaccines substantially improves avidity of the gE-specific antibodies. In addition, induction of neutralizing antibodies is superior after vaccination with the recombinant vaccine. In summary, the quality of gE-specific antibodies seems to be higher after vaccination with the recombinant vaccine. The exact role of these highly functional antibodies for protection remains to be elucidated [129].

### Clinical efficacy and effectiveness

Vaccine efficacy in persons older than 60 years was 51% against HZ and 67% against PHN in the pivotal Phase III randomized, double-blinded, placebo-controlled trial for the live-attenuated vaccine [130]. However, as mentioned above reduced T cell responses were observed in the oldest participants, which was also reflected in lower efficacy (60-69y: 64%; 70-79y: 41%; ≥80y: below 20%) in the pivotal trial and in an additional study, which showed higher efficacy of 70% in a younger cohort aged 50-59 years [122, 131]. The protective effect of the vaccine waned over time and was lost approximately 10 years after vaccination [132], but re-vaccination after 10 years results in a booster effect and restores immune responses [133].

The two pivotal phase III randomized, placebo-controlled clinical trials for the recombinant, adjuvanted vaccine included more than 30,000 participants older than 50 or 70 years, respectively [134, 135]. No major safety concerns were identified after two doses of vaccine administered 8 weeks apart. The majority of adverse effects were temporary reactions at the site of infection and systemic symptoms, such as headache, myalgia or fatigue were relatively frequent, but mild. Clinical efficacy against HZ was 97.2% (95% CI: 93.7-99.0) in persons over 50 and 89.8% (95% CI: 86.8-94.5) in persons over 70 years of age, respectively in the two phase III trials. Combined analysis of all participants did not reveal statistically significant differences between age groups 70-79 and ≥80 years [134, 135]. Despite a slight decline, efficacy remained above 85% for the first four years after vaccination and did not decrease further until 7 years post-vaccination [136]. Post-herpetic neuralgia was very rare in the vaccinated study cohort, with no cases in participants younger than 70 years and a vaccine efficacy of 88.8% (95% CI: 68.7-97.1) in persons older than 70 [134].

Frailty status was determined for more than 90% of the participants of the two studies, and as expected, prevalence of frailty increased with age. Vaccine efficacy against HZ was above 90% also in the frail and pre-frail sub-cohorts [137]. Vaccine effectiveness was lower in an observational post-licensure study, namely 70.1% (95%CI: 68.6-71.5), but did also not decline with age (65-79y vs. ≥80y). This study showed that the second dose is required for optimal protection, but that a delay to administer the second dose (>180 days after dose 1) does not impair protection [138].

In contrast to the live-attenuated vaccine, the recombinant vaccine is suitable for immunocompromised patients. Safety and immunogenicity have been demonstrated in patients after renal transplantation [139], in HIV-positive patients [140] and in patients receiving chemotherapy or immunosuppressive treatment for hematologic malignancies [141].

### Future developments

A clear limitation of the live-attenuated vaccine is that its use is contraindicated for patients under immunosuppression. An inactivated vaccine, which used a heat-inactivated formulation of the Oka Merck virus strain demonstrated 63.8% (95%CI: 48.4-74.6) and 83.7% (95%CI: 44.6-95.2) efficacy against HZ or PHN, respectively in a Phase III trial in hematopoietic stem-cell transplant recipients [142]. However, four doses are required, and with the licensure of the recombinant, adjuvanted vaccine (see above), which is also suitable for immunocompromised patients it seems unclear how this vaccine will be used. There is also ongoing development of an alternative subunit adjuvanted vaccine (NCT03820414).

## COVID-19

In the current SARS-CoV-2 pandemic older adults, persons with underlying co-morbidities and obese individuals have the highest risk for severe disease and death from COVID-19 [143–145]. Reports of long-lasting health sequelae after recovery from COVID-19 (“long COVID”) are accumulating [146, 147], but this complication seems to be more frequent in younger patients. Efficient vaccines are essential to control the pandemic and older adults are an important target group for vaccination against SARS-CoV-2. Most national vaccination programs prioritized old adults and this age group was among the first recipients of SARS-CoV-2 vaccines. Several vaccines against SARS-CoV-2 have been licensed in different countries, clinical trials with additional vaccine candidates are ongoing and a plethora of vaccine candidates are in pre-clinical development [148]. This summary focusses on the SARS-CoV-2 vaccines currently licensed in Europe, namely the mRNA-based vaccines of BioNTech/Pfizer (Comirnaty) and Moderna (Spikevax) and the adenoviral vectors of AstraZeneca (chimpanzee adenovirus ChAdOx1; Vaxzevria) and Johnson&Johnson/Janssen (human Adenovirus-26; COVID-19 Vaccine Janssen) and highlights their safety, immunogenicity, efficacy and effectiveness in older adults. With the exception of Vaxzevria, they are also licensed for emergency use in the US. All four vaccines deliver genetic information for the SARS-CoV-2 spike protein, which is then produced by cells of the vaccinee. Comirnaty, Spikevax and Vaxzevria are administered in a two-dose regimen. COVID-19 vaccine Janssen is a single-dose vaccine. A complete overview of SARS-CoV-2 vaccine development is beyond the scope of this article and can be found elsewhere [149].

### Immunogenicity

First-in-human clinical trials are usually performed in young, healthy adults, but for SARS-CoV-2 vaccines clinical development moved quickly towards the inclusion of older adults. As an example, the phase I dose escalation trial for Spikevax was extended from the original cohort (18-55 years) to also include participants >55 and >70 years [150] and Comirnaty was also tested early in the age groups 18-55 and 65-85 years [151]. Both studies demonstrated similar immunogenicity for the different age groups. The phase I/IIa study for COVID-19 vaccine Janssen included adults aged 18-55 or more than 65 years, respectively. Antibody and CD4^+^ T cell responses after one dose of vaccine were slightly lower in the older age group. The age-associated decrease in immunogenicity was more pronounced for CD8^+^ T cell responses, with cytokine-producing T cells detectable in 51% of the younger and 36% of the older cohort, respectively [152]. Phase II and III studies of all vaccines also investigated immunogenicity and confirmed these results. Antibody levels and neutralizing titers, as well as T cell responses after vaccination with Vaxzevria were similar in the age groups 18-55y, 56-69y, and ≥70y [153]. Further immunogenicity trials including older adults are still ongoing for the different vaccines, and now also include the newly emerging virus variants. Neutralizing antibodies against the original virus after the first dose of BioNTech/Pfizer mRNA vaccine are substantially lower in persons older than 80 years compared to younger age-groups, and this effect is even more pronounced for virus variants. However, the age-associated differences were less prominent after completion of the two-dose vaccination series. In addition, differences in B cell memory, somatic hypermutation and T cell responses were observed comparing persons younger or older than 80 years [154].

Safety of all vaccines has been investigated extensively. This summary highlights age-related aspects and therefore mainly relies on the safety data from later stage trails, which included more older participants. The safety profile of the two mRNA vaccines Comirnaty and Spikevax are similar, with local pain at the site of injection being more frequent than redness and swelling. Fatigue, headache, muscle pain and chills are the most frequent systemic events [155, 156]. The reactogenicity profiles of the vector vaccines Vaxzevria and COVID-19 vaccine Janssen are similar, with pain and tenderness reported as the most common local and fatigue and headache as the most common systemic reactions [152, 153, 157]. Local and systemic reactogenicity was higher after the second than after the first dose of mRNA vaccines, but higher after the first dose of Vaxzevria, compared to the second dose of this vaccine. For all vaccines, reactogenicity was lower in older compared to younger adults. Severe adverse events were rare for all vaccines and mostly not vaccine related. After introduction of the vaccines and use in a large number of persons very rare severe adverse events were observed including allergic reactions, mainly against the mRNA vaccines [158, 159] and thrombosis with thrombocytopenia syndrome (TTS) after vaccination with the adenoviral vector vaccines [160]. Rare cases of myocarditis have been reported in adolescents and young adults (<30y) after vaccination with mRNA vaccines [161]. These rare complications are more relevant for younger adults and will therefore not be discussed in detail.

### Clinical efficacy and effectiveness

Phase III clinical trials to determine vaccine efficacy included older adults, but particularly for the oldest age groups the number of participants was usually not sufficient for statistical analysis. For the BioNTech/Pfizer vaccine, overall efficacy against symptomatic SARS-CoV-2 infection was 95.0 % (95%CI: 90.0-97.9) after 2 doses with a 3-week interval. Vaccine efficacy did not differ in the age groups 16-55y, >55y, and ≥65y. However, analysis of the oldest age group (≥75y) was not statistically significant with 5 cases of COVID-19 in the placebo and zero cases in the vaccinated cohort, respectively. In this study more than 40% of the participants were older than 55 years, but less than 5% were over 70 years old [156]. Results were similar for the second mRNA vaccine, Spikevax. Approximately 25% of the participants in this pivotal phase III study were older than 65 years. The vaccine interval was 4 weeks between the two doses and overall efficacy against symptomatic SARS-CoV-2 infection was 94.1% (95%CI: 89.3-96.8) after the second dose. For the different age groups efficacy was 95.6% (95%CI: 90.6-97.9) and 86.4% (95% CI: 61.4-95.2) for participants younger or older than 65 years, respectively [155]. Additional analyses showed an efficacy of 82.4% (95%CI: 46.9-93.9) for persons aged 65-75y, and again no statistically valid results for the subgroup older than 75 years of age, as no COVID-19 cases occurred in the vaccinated group versus 7 cases in the placebo group [162]. Clinical trial results for Vaxzevria were reported as a summary of several studies with differences in the age of participants, dosing of the vaccine and time intervals between the two doses. After two full-dose vaccinations, clinical efficacy against symptomatic SARS-CoV-2 infection was 62.1% (95%CI: 41.0-75.7). Only 12% of the participants were older than 55 years and no age-stratified sub-analysis was provided [163]. As a result, some countries recommended this vaccine only for younger adults at the beginning of their vaccination campaigns. In March 2021, press releases announced 79.9% efficacy against symptomatic disease in persons older than 65 years for an ongoing Phase III trial in the US, but the full data are not published yet (NCT04516746; [164]). In the pivotal trial for the single-dose COVID-19 vaccine Janssen clinical efficacy against moderate to severe COVID-19 disease was reported to be 66.1% (95% CI: 55.0-74.8). There were no differences between the age groups (younger or older than 60 years) with 33% of the participants falling into the older age group [152]. Vaccine efficacy against asymptomatic infection is of particular interest from a public health perspective, because potential transmission of the virus from vaccinated, asymptomatically infected individuals might play an important role in overall spread of the virus. In the phase III trials, different strategies were implemented to detect asymptomatic infections. A sub-cohort of the participants in the Vaxzevria studies was monitored for asymptomatic infection by weekly self-swabs and PCR testing, but efficacy was not statistically significant after receiving two full-dose vaccinations [163]. In contrast, the investigators of the COVID-19 vaccine Janssen determined antibodies against the viral N protein 71 days after the vaccination. As this antigen is not present in the vaccine, this assay identifies persons who had been infected. Vaccine efficacy against asymptomatic infection was 65.5% (95%CI: 39.9-81.1) in this preliminary analysis [152]. The phase III trials with the two mRNA vaccines announced to provide similar seroconversion data after a longer observation period.

Due to the limited supply of vaccines at the beginning of national vaccination campaigns specific target groups including health care workers and older adults were prioritized and were the first to receive the vaccine. Effectiveness in “real life” has been and is still monitored in many countries. Asymptomatic infections were mainly monitored in health care workers as they are routinely tested for active infection irrespective of vaccination. High effectiveness of the mRNA vaccines was demonstrated already after the first dose and further increased after full vaccination [165, 166]. However, these studies did not provide data for older adults. Nation-wide efficacy data was first available from Israel, where the vaccination campaign with Comirnaty was rolled out extraordinarily fast after licensure. Vaccine effectiveness was reported against asymptomatic and symptomatic SARS-CoV-2 infection, as well as for COVID-19 related hospitalization and death for several age groups (16-44y; 45-64y; ≥65y). The lowest effectiveness was 85.9% (95%CI: 80.2-89.9) for asymptomatic infection in the oldest age group, all other outcomes were higher, most of them above 95% [167]. The oldest age group was further stratified, but no differences were observed between the age groups ≥65y, ≥75y, and ≥85y. These findings demonstrated excellent effectiveness against infection and disease in all age groups, including the very old. A Danish pre-print reported a vaccine effectiveness against PCR-confirmed SARS-CoV-2 infection of 64% (95%CI: 14-84) in long-term care facility residents (median age 84y) and 90% (95%CI: 82-95) in health care workers (median age 48y) more than 7 days after the second dose of Comirnaty. It has to be pointed out, that this study investigated PCR-confirmed infections, not symptomatic disease [168]. A study in Spanish long-term care facility residents provided vaccine effectiveness estimates of 71% (95% CI: 56–82%), 88% (95% CI: 75–95%), and 97% (95% CI: 92-99%), against SARS-CoV-2 infections (symptomatic and asymptomatic), COVID-19 hospitalizations and deaths, respectively. In this study both mRNA vaccines were included [169]. Additional effectiveness studies were performed in other countries and also with the adenovector vaccines, mostly confirming these results. A comprehensive review of vaccine efficacy and effectiveness can be found elsewhere [170].

### Future developments

Many factors are relevant for optimal protection. There is evidence that longer time-intervals between the two vaccine doses are beneficial [171, 172] and that heterologous prime-boost schedules might induce superior immune responses [173], but most of these studies are too small to determine clinical efficacy of the different schedules. Duration of protection and as a consequence the need and optimal timing for additional vaccines doses are currently a major focus of interest. First reports are emerging that antibody and T cell responses persist for several months after vaccination [174, 175], but it is unclear how long clinical protection lasts. It is impossible to study this question in the context of the placebo-controlled clinical trials as in the meantime vaccination was offered to the participants who originally received placebo. In July 2021 Israel reported that they observed only 64% vaccine effectiveness against infection and symptomatic infection in the previous weeks (compared to>90% in the early reports; see above), while effectiveness against serious illness and hospitalization was still 93% [176]. However, so far no scientific publications are available confirming these data. Israel decided to offer a third vaccine dose to persons older than 60 years, who received their second dose at least 5 months earlier, extending this recommendation soon thereafter to everybody older than 50, and then 40 years. Other countries, e.g. Germany also plan to administer third doses to high-risk individuals, e.g. older adults starting in autumn 2021, while US authorities do not support a third dose for immunocompetent individuals at the moment, but prepare for the possibility that the need arises. At the same time, WHO calls for halting administration of third doses at a time when a large proportion of the world´s population have not even received a first dose. This discussion is further complicated by the fact that different virus variants arose over time and have to be taken into account. At the time of the first clinical trials the “original” virus was gradually replaced by the alpha variant (B.1.117) and at the moment almost all cases e.g. in Europe and the US are caused by the delta variant (B1.617.2). Vaccine effectiveness differs for the variants, particularly after the first dose [177], and therefore direct comparisons of effectiveness at different time points after vaccination are difficult. It is important to monitor long-term protection specifically in high-risk populations, such as older adults, to make evidence-based decisions. Modified vaccines, which incorporate the crucial changes in the viral genome are being developed.

In future years, SARS-CoV-2 vaccines might become part of national vaccination schedules for everybody, or at least for risk groups. First studies are ongoing investigating co-administration of SARS-CoV-2 vaccines with other vaccines, such as influenza or pneumococcal vaccines (NCT04848467, NCT04790851), and development of combination vaccines (e.g. SARS-CoV-2 and influenza) has started [178].

## Vaccines for all adults

Many countries recommend regular booster vaccinations against tetanus and diphtheria, sometimes in combination with acellular pertussis and/or inactivated polio for all adults. Other vaccines might be relevant in some countries, e.g. against tick-borne encephalitis in endemic areas. Most recommendations for these vaccines do not mention older adults in particular, but some countries e.g. in Europe Austria, Liechtenstein, France and Portugal advocate shortened booster intervals for older adults. Tetanus- and even more so diphtheria-specific antibody concentrations are frequently below the levels considered to be protective for adults, and are even lower in older age groups [179–183]. In one of our studies in Austria, booster shots with a combined vaccine containing diphtheria toxoid did not provide long-term protection in almost half of the older participants [180, 184]. Tetanus- and diphtheria- specific antibody levels vary greatly in different European countries. In general, protection against tetanus is adequate in most countries, whereas antibodies against diphtheria are below the protective level for a substantial fraction of the population in some countries, and are decreasing with age [185]. A detailed overview on vaccination against tetanus and diphtheria can be found elsewhere [186]. Antibody responses after booster vaccination against tick-borne encephalitis are also lower in old compared to young adults and decline over time [187, 188]. Pertussis infection can be severe in older adults and increased numbers of cases have been observed in the last years in this age group [189–191]. To prevent not only severe cases in adults but also transmission to newborns, who are too young to be vaccinated, vaccination against pertussis is important for adults. Regular booster doses of Tdap (tetanus, diphtheria, acellular pertussis) vaccine are well tolerated and immunogenic in young and older adults, but antibody concentrations are lower in the older age groups [179, 192]. However, only few countries recommend regular booster immunization with combination vaccines containing the pertussis component and some recommend one booster dose during adulthood.

As mobility, financial resources and health of older adults have improved over the last decades, long-distance travel and as a consequence the need for travel vaccines have become increasingly common for this age group. Despite the fact that some tropical diseases, e.g. Japanese encephalitis and typhoid fever are more frequent and severe in older adults [193, 194], only limited data are available on immunogenicity and efficacy of travel vaccines in older age groups. Immunization guidelines rely primarily on studies with young adults. A detailed review of travel vaccines for older adults is beyond the scope of this article and can be found elsewhere [195, 196]. The loss of naïve T and B cells is a hallmark of immunosenescence, and an impaired generation of memory responses has been reported in aged animals [197, 198]. This suggests that responses to neo-antigens, such as travel vaccines, might be particularly impaired in old age. Antibody responses to Hepatitis A and B vaccination are already lower in middle-aged adults and non-responders to Hepatitis B vaccine are more frequent in older age groups [199–202]. It has to be emphasized that vaccination against Hepatitis B is relevant for older adults not only as a travel vaccine, but also in other settings, such as for hemodialysis patients and household contacts of infected patients. Immunogenicity and efficacy of the live-attenuated yellow fever vaccine is high even in older adults, but the risk for rare severe adverse events increases with age. Yellow-fever vaccine-associated viscerotropic disease mimics viral infection and has a mortality of up to 60% [203]. A yellow fever vaccine with an improved safety profile in the older population would be desirable.

## Conclusions and Outlook

Older adults are at increased risk for severe disease caused by various pathogens and this is particularly evident in the current SARS-CoV-2 pandemic. The last 18 months have also shown that novel vaccines can successfully be developed in a short period of time. Clinical efficacy of COVID-19 vaccines is high, even in older adults. Nevertheless, it seems that immunity and potentially protection wanes faster in older age groups and that additional doses will be required earlier in these cohorts highlighting the importance of including specific age- and risk-groups in clinical and observational studies. Several vaccines against other pathogens, such as influenza, *S. pneumoniae* and herpes zoster are available for older adults, and vaccines that are recommended for all adults (e.g. diphtheria, tetanus, pertussis) are also relevant in old age. Modifications of the vaccines, such as higher antigen dose or adjuvants for influenza vaccines, and optimization of vaccination schedules are strategies to improve vaccine effectiveness for older adults. However, vaccination coverage is still unsatisfactory for many of these vaccines and systematic data on vaccine uptake are not collected in all countries. Barriers to vaccine uptake are manifold and include lack of access, which can be caused by financial or other constraints, and personal decisions against vaccination based on missing information or misinformation regarding risk of disease and benefit of vaccination. To overcome these issues documentation of vaccine uptake in conjunction with data such as age and other risk factors is crucial in order to identify gaps in coverage and develop strategies to specifically target the relevant cohorts. Vaccination documentation is important also on a per-person scale in order to deliver vaccines at the right time and to enable reminder systems.

There are still many pathogens, which cause high morbidity and mortality in the older population, for which no vaccines are available, but would be highly desirable. The risk for nosocomial infections is high for older persons due to an increased frequency of invasive procedures, hospitalization or long-term care, and antibiotic resistance of bacterial pathogens is an increasing problem in these settings. *Staphylococcus aureus* and *Escherichia coli*, which are responsible for infections of catheters, prostheses, or surgical wounds and *Clostridium difficile*, which is the most common cause of nosocomial diarrhea are among the most relevant bacterial nosocomial pathogens [204]. Several potential vaccines have been clinically tested in different settings, but did not fulfil expectations, and more recent vaccine candidates are still in pre-clinical development. Vaccines against other pathogens, such as *Klebsiella pneumoniae*, *Pseudomonas aeruginosa*, *Acinetobacter* ssp*.*, and *Candida* spp. could have a substantial impact. Norovirus is the most relevant viral nosocomial infection and outbreaks in hospitals and long-term care facilities are frequent. The disease is characterized by vomiting and diarrhea and can be severe in older adults. In 2016 the World Health Organization stated that the development of a norovirus vaccine should be considered an absolute priority, and vaccine development is ongoing [205]. A detailed summary on vaccines for nosocomial infections of older adults has recently been published [206]. Respiratory syncytial virus (RSV) causes severe respiratory infections in infants, but older, particularly frail persons are also at high risk for severe disease. It is estimated that 18,000 hospitalizations and 8,400 deaths per year are caused by RSV in the UK, and most of these cases occur in the older population [207]. RSV vaccine development was impeded by the fact that the first RSV vaccine in the 1960s was associated with a risk for antibody-mediated disease enhancement in infants. Several novel vaccine candidates have been in clinical trials over the last years, but failed to demonstrate clinical efficacy despite encouraging safety and immunogenicity data [208]. There are many more vaccine candidates against a plethora of pathogens in various stages of development, but a complete overview is beyond the scope of this review.

Optimization of existing vaccines and vaccination strategies as well as development of novel vaccines for “old” (e.g. universal influenza or pneumococcal vaccines) and “new” pathogens have the potential to substantially improve health and quality of life in older adults. A detailed knowledge about age-associated changes of the immune system is essential in order to rationally design vaccines which hopefully overcome these limitations, and it is of utmost importance to consider age-related aspects already early in the vaccine development process (Table 3).

**Table 3:** Challenges of vaccine development for older adults

**Limitations of current vaccines for older adults**	
• Age-dependent decline of immunogenicity and clinical effectiveness of several vaccines	
• Potentially more rapid waning of immune responses and protection with age	
• Lack of vaccines for relevant pathogens	
• Unsatisfactory vaccination coverage	
**Strategies to overcome these limitations**	
• Modification of vaccines (e.g. higher antigen dose, adjuvants)	
• Optimization of vaccination schedules	
• Improved vaccination documentation and access to vaccination	
**Remaining challenges**	
• Development of universal influenza and pneumococcal vaccines	
• Development of vaccines against nosocomial and other relevant pathogens	
• Rational design of vaccines to specifically overcome age-related immunological limitations	

## Data Availability

Not applicable
